# Selections and Abstracts

**Published:** 1916-05

**Authors:** 


### SELECTIONS AND ABSTRACTS

### ARMY MEDICAL CORPS EXAMINATIONS.

The Surgeon General of the Army announces that preliminary examinations for the appointment of First Lieutenants in the Army Medical Corps will be held on July 17, 1916 and August 14, 1916, at points to be hereafter designated.

Full information concerning these examinations can be procured upon application to the "Surgeon General, U. S. Army, Washington, D. C." The essential requirements to securing an invitation are that the applicant shall be a citizen of the United States, shall be between twenty-two and thirty years of age, a graduate of a medical school legally authorized to confer the degree of Doctor of Medicine, shall be of good moral character and habits, and shall have had at least one year's hospital training as an interne, after graduation. The examinations will be held simultaneously throughout the country at points where boards can be convened. Due consideration will be given to localities frim which applications are received in order to lessen the traveling expenses of applicants as much as possible.

In order to perfect all necessary arrangements for the examination, applications must be completed and in possession of the Adjutant General, at least three weeks before the date of examination. Early attention is therefore enjoined upon all intending applicants. There will be more than one hundred vacancies to be filled after July 1st, when the Bill for the reorganization of the Army becomes a law.

---

### DO YOU KNOW THAT

Light promotes cleanliness?

A clean mouth is essential to good health?

Physical training in childhood is the foundation of adult health?

The U. S. Public Health Service issues publications on hygiene and sanitation for free distribution?

Isolation is the most efficient means of controlling leprosy?

Headache is nature's warning that the human machine is running badly?

Bullets may kill thousands—flies tens of thousands?

Obesity menaces longevity.

---

### STATE HEALTH DEPARTMENTS FIGHTING CANCER

Among the many agencies now active in the campaign against cancer, several of the most progressive state boards of health are making notable efforts to spread the gospel of hope which is found in the early recognition of the danger signals of the disease and its prompt and competent treatment. The health authorities of Massachusetts, New Hampshire, Ohio, Indiana, Michigan Virginia, North Carolina, Kentucky, West Virginia, and Idaho have been especially active in disseminating trustworthy information and advice about the prevention and cure of cancer.

The New York State Health Department, under the leadership of Commissioner Hermann M. Biggs, is the latest to enlist its forces in the war against cancer. The entire March number of "Health News," the Department's Monthly Bulletin, is devoted to consideration of the nature, prevalence and treatment of malignant disease with the object of creating among the people a "healthy vigilance which leads to the taking of expert advice on the first appearance of danger signals."

"There is nothing that any one of us can do to prevent the occurrence of cancer except in avoiding certain specified causes of local irritation," says "Health News," in an editorial which opens the discussion. "On the other hand, there is incontrovertible testimony as to the probability of its cure in a large percentage of cases if taken in time. That cure consists in the complete surgical removal of the growth at the earliest possible moment. Early diagnosis, early removal—there is not now nor has there ever been any other successful method of curing the disease."

The leading article in this special issue of the Health Department's Magazine is by Dr. Francis Carter Wood, Director of Cancer Research at Columbia University. Additional papers are contributed by other notable figures in the scientific world, including Frederick L. Hoffman, LL.D., statistician of the Prudential Insurance Company and Chairman of the Statistical Advisory Board of the American Society for the Control of Cancer, and Dr. Harvey R. Gaylord, Director of the New York State Institute for the Study of Malignant Disease.

Writing on "What People Should Know About Cancer," Dr. Wood endeavors to dispel some of the mistaken popular notions which have grown up regarding this disease. He disposes of the stories regarding "cancer villages," "cancer houses," or "cancer belts," briefly showing that the occurrence of a number of cases in a house usually is due to the fact that the occupants are old people; that "cancer villages' usually are small towns from which most of the young people have migrated, and that in like manner "cancer belts" are found to be sections of the country where the population is distinctly aged.

The idea that cancer is hereditary is likewise made light of by Dr. Wood and he declares that there is no reason whatever to worry because one member of a family has suffered from the disease. "It does not at all follow that any other member of a family will have it," says Dr. Wood, and quotes from the laws governing statistics to show that if there are two or more cases in a family it is due purely to chance.

The quackery which is practiced by unscrupulous people in the treatment of cancer is severely censured both by Dr. Biggs and by Dr. Wood. It is made perfectly plain that cancer is comparatively easy to cure if it can be taken in time. The Bulletin declares that if the simple truth is thoroughly established that cancer begins i ncomparatively innocent form and in most instances in a recognizable form, it can be successfully combated. Dr. Hoffman in his paper emphasizes "the supreme importance of the earliest possible diagnosis and the incalculable value of the earliest possible medical and surgical treatment." Dr. Wood puts stress on the declaration that if the disease can be diagnosed in its early stage, the cancer can be removed with very great possibilities as to permanent cure. "The Commissioner of Health takes this opportunity, says Dr. Biggs, "to warn the people of the state against the expenditure of money—often ill-afforded—the raising of false hopes and, above all, the waste of precious time through the use of alleged cancer cures and consultation with their unscrupulous purveyors."

In anticipation of a popular demand for information regarding cancer, a large edition of the "Health News" for March has been printed. Any one who desires the full information as contained in the magazine may secure a copy of the publication, free of charge, by addressing the State Department of Health at Albany, N. Y."

---

### DO YOU KNOW THAT

Life is a constant struggle against death?

Dirty refrigerators may make sickness?

The U. S. Public Health Service issues free bulletins on rural sanitation?

The defective citizen of today is ofttimes the unhealthy child of yesterday?

Every man is the architect of his own health?

Its the baby that lives that counts?

Tuberculosis is contagious, preventable, curable?

The full dinner pail—the open window—the clean well—make for health?

---

### UNITED STATES CIVIL SERVICE EXAMINATION.

**Clinical Director (Male), $2,000, Government Hospital For the Insane, June 27, 1916.**

**The United States Civil Service Commission announced an open competitive examination for clinical director, for men only. From the register of eligibles resulting from this examination certifiaction will be made to fill a vacancy in this po-**

sition in the Government Hospital for the Insane, Washington, D. C., at a salary of $2,000 per annum, with maintenance in the hospital, and vacancies as they may occur in positions requiring similar qualifications, unless it is found to be in the interest of the service to fill any vacancy by reinstatement, transfer, or promotion.

Competitors will not be assembled for examination, but will be rated on the following subjects, which will have the relative weights indicated: Subjects: 1, Education, weight, 40; 2, Training and experience, weight, 60; total weight, 100.

Applicants must be graduates of a medical school of recognized standing, and must have been in actual charge of a medical service in an institution for the care and treatment of the insane for at least five years, during the entire period of which time they must have received special training in psychiatry. They must have contributed to medical literature (a) on the general subject of insanity in some of its phases, or (b) by report of cases coming under their observation.

The appointee, in his capacity as clinical director, will have general administrative supervision of the medical work of the hospital together with supervision and care of the hydrotherapeutic departments, operating room, and training school for nurses; all transfers of patients from one service to another will be made through him; he will have charge of the clinical records and will see that they are properly kept, and be in a position to offer suggestions that recent medical literature may contain along the lines of clinical psychiatry.

Statements as to education, training, and experience, are accepted subject to verification.

Applicants must not have reached their thirty-fifth birthday on the date of the examination.

This examination is open to all men who are citizens of the United States and who meet the requirements.

Persons who meet the requirements and desire this examination should at once apply for Form 1312, stating the title of the examination desired, to the United States Civil Service Board, Post Office, Boston, Mass., Philadelphia, Pa., Atlanta, Ga., Cincinnati, Ohio, Chicago, Ill., St. Paul, Minn., Seattle, Wash., San Francisco, Cal.; Customhouse, New York, N. Y., New Orleans, La., Honolulu, Hawaii; Old Customhouse, St. Louis, Mo.; Administration Building, Balboa Heights, Canal Zone; or to the Chairman of the Porto Rican Civil Service Commission, San Juan, P. R. Applications should be properly executed, excluding the medical and county officer's certificates, and must be filed with the Commission at Washington prior to the hour of closing business on June 27, 1916.

Issued May 23, 1916.

---

### MILITARY PREPAREDNESS FROM A MEDICAL STANDPOINT.

FOR THE REGIMENTAL SANITARY DETACHMENT BY MAJOR EDMUND FOWLER, NEW YORK

Surgeon, Seventh Regiment Infantry, N. G. N. Y.

(New York Medical Journal, March 23, 1916.)

The regimental medical department and the medical departments of other troop units are responsible for the execution of sanitary measures and for the temporary care, treatment, and transportation of the regimental sick and wounded. The regimental surgeon is the adviser of the regimental commanding officer in medical and sanitary matters, and the entire regimental personnel is instructed by him in personal hygiene and first aid.

He is held responsible for the records, reports, and returns, and any deficiencies in supplies and equipment for his department, and for the provision of a serviceable first aid packet for every man of his regiment. For the early detection of disease he physically examines every soldier and civilian with the regiment monthly, and on the march and in camp makes frequent examination of the sick in order to insure their proper treatment and disposal.

The work of the regimental sanitary detachment, augmented if need be by civilian labor, or by details from the line, and the first aid during an engagement are under the supervision of the regimental surgeon. In addition to its other duties, the detachment procures, or improvises field sanitary apparatus, prepares and applies disinfectants supervises the water supply and its purification, the sanitation of kitchens and cooking places, the preparation, care, and filling in or latrines and urinals, and the marking of old sites thereof; the collection, removal, and disposal of wastes, solid and liquid; and the policing of washing and bathing places.

The equipment should include hospital stores, quartermaster ordinance, and subsistence property; and be complete for a regimental infirmary, the ordinary provision during field service for the care of the trivially sick in camp. The very ill, being an incumbrance to a command in the event of a move, are promptly transferred according to orders from the chief surgeon division.

In combat the supplies of the regimental medical personnel consist only of those carried on their persons, the hand litters, and the cases of surgical dressings upon the pack animal. The pack animal (constituting the sanitary combat train) is the sole transportation for the regimental aid station.

During an engagement before the aid station is established the entire regimental medical personnel accompany the troops, treating the wounded wherever found and pausing only to give appropriate first aid. When later in the combat the number of the wounded constantly requires several attendants, the surgeon orders the establishment of the regimental aid station, as near the firing line and as much under shelter as possible. No elaborate arrangements obtain at the aid station. It is mainly a place for assembling the wounded, applying first air, readjusting dressings, stopping hemorrhages, immobilizing fractures, and administering restoratives and analgesics when necessary. If practicable, stimulating food and drink should be prepared. The wounded are directed to the rear and, if unable to walk, are carried to the dressing station or possibly to the field hospital by the bearers sent forward by an ambulance company. The aid station is cleared, and the wounded in advanced positions are reached by the regimental and ambulance company personnel, especially during darkness. Lieutenant Hutton has prepared a chart showing graphically the scheme of medical transport service at the front, but I can only mention this subject here. Many important problems present themselves in this field of operations.

In order that the duties outlined above may be efficiently performed, it is apparent that the officers and men of the medical units must be proficient, not only in their special work, but also as trained soldiers. Without discipline and a knowledge of military organization, regulations, and administration, it is impossible for the most skilled physician or surgeon to organize, train, control, or use his personnel to advantage. Likewise, with the medical units of the rear and base, the medical personnel must be properly prepared to be efficient.

It therefore becomes our duty, if we are loyal citizens, to take up seriously the problem of military preparedness from a medical standpoint and to assist the army in its ceaseless endeavors to get ready for the inevitable.

For those who would go to the front with the fighting troops (the younger men), there are at present practically but two ways by which these may orient and partially train themselves for duty, namely, by service in the National Guard, or in the field with the Medical Reserve Corps. Training with troops is imperative because, as we physicians know, theory is no criterion of efficiency. For the older men there is at present no method available.

Of course, old and young alike, every one of us, owe to our country our personal support and service, and whenever called upon, the medical profession must not be found wanting. But unless we organize, tabulate, and classify our medical resources and assign to each medical institution and its personnel definite preparation, when war or other terrible national calamities come, as in the past, we shall not be ready to play the game.

If by bringing to my readers' attention, conditions abroad and our endeavors and shortcomings at home, this paper has awakaned in their minds only a sense of unpreparedness, it will have been a miserable failure. If, on the other hand, it is instrumental in arousing individual and collective action to the end that we prepare for war, then it will have accomplished its purpose.

In a democracy, it is eminently fitting that every man give to his country his services when war threatens. How more fitting, then, that in peace he equip himself to meet any external aggression. It is not the duty of a few to protect all. The service must be universal. Surely it is better for every one to give a little time to preparedness now, than to wait for trouble, and then not only give one's whole time, but it may be almost uselessly one's life.

616 Madison Avenue.

---

### THYMOL FROM HORSEMINT.

### Government Specialists Find Commercial Possibilities in Development of New Industry.

Washington, D. C.—That the commercial production in this country of thymol from horsemint may be, under favorable circumstances, a profitable undertaking is indicated by the recent investigations of the U. S. Department of Agriculture, the results of which are published in Bulletin 372. Thymol is extensively used in medicine and forms the basis of a number of important pharmaceutical compounds. In the past it has been imported from northern Europe where it is manufactured from ajowan seed grown in northern India. Now that the European war has reduced these importations from overa 18,000 pounds in 1914 to a little more than 2,000 in 1915, it is believed that to some extent the demand can be supplied at home. For several years the Department of Agriculture has been conducting experiments with horsemint which occurs as a common weed in many localities. These experiments have resulted in improving the plants by selection to a point which it is said warrants the use of horsemint for the commercial production of thymol.

Horsemint is found wild on light sandy soils over the entire region from southern New York to Florida and westward to

Wisconsin, Kansas and Texas. It is probable that it will thrive under cultivation wherever it is found growing wild, but local economic conditions must be considered in determining whether or not its production would be profitable. The investigations of the Department of Agriculture indicate that by distilling the improved plants an average of 20 pounds of oil per acre may be obtained from first-year plantings, and that in succeeding years the yields should be at least 30 pounds per acre. The phenol content of this oil may be assumed to be about 70 per cent., almost all of which is thymol. The yield of thymol per acre of horsemint, therefore, should be for the first year a little less than 13 pounds, and for suceeding years a little less than 20 pounds. At the average price of thymol for a number of years has been about $2 a pound, the gross returns per acre from a horsemint plantation are estimated in the bulletin already mentioned, at about $25.72 for the first year, and $38.58 for each suceeding year.

It is more difficult to estimate with accuracy the cost of producing the thymol. In the opinion of the investigators it is doubtful whether the profits from the industry will be sufficient to warrant anyone in engaging in it unless the horsemint is grown in connection with other oil-yielding plants for which a distilling apparatus is required. In that even, of course, the entire cost of the distilling plant can not be charged against the thymol industry alone. For this reason in the estimates of cost production published in Bulletin 372 such items as land rent, taxes, depreciation, upkeep, and interest on the distilling plant have not been included. Excluding these items it is believed that thymol can be produced at an approximate cost of $23 per acre the first year, and $19 per acre thereafter. This figure includes the growing of the plants, fertilizer, cultivation, harvesting, and distilling. A plantation of horsemint will not have to be replanted oftener than once in five years and under average conditions may continue to give a good yield for a still longer time. After the first year a material reduction can be made in the cost of fertilizers if the distilled herb is returned to the soil. These facts account for the reduction in the cost of production after the first year.

Horsemint seed matures in the Southeastern States during August and September and is ready to be gathered as soon as the calyx is dry and has assumed a dark-brown color. The entire heads can readily be stripped off by hand. They should be spread out on a cloth or tight floor and thoroughly dried. The seed can then be removed by rubbing through a sieve, common window screening being about the right size. Where the winters are free from severe frost and snow, as in the extreme Southeastern States, the best results can be secured by planting the seed about the first of September in a carefully prepared seed bed. About two month after sowing, when the plants are about 2 inches high, they are ready for transplanting to the fields. Fuller information in regard to methods of cultivation, harvesting, and distilling are contained in Bulletin 372.

---

### DISEASE IN APPARENTLY HEALTHY COLORED GIRLS.

An Investigation, by Gordon Wilson, M. D., Baltimore. Professor of the Principles of Medicine, University of Maryland; Physician in Charge, Municipal Tuberculosis Hospital.

(New York Medical Journal, March 23, 1916.)

At the request of the supervisors of the City Charities of Baltimore City, Dr. Mary Hodge and I made an examination of a numbe of colored girls who were minor wards of the city, residing in a reformatory institution which received its inmates not only from the city but also from the counties. This report is based on the examination of seventy-six of these colored girls, committed from the city to this institution. I want to take this opportunity of thanking Dr. Mary Hodge, of the Johns Hopkins dispensary staff, for permission to use the results of her examinations in this paper.

Before undertaking the examination of the inmates, a method was decided upon which included taking the weight, height, as well as the age of the girl to be examined a thorough physical examination, a Wassermann test and the examination of smears made from the urethra or vagina.

The girls were dressed for the examination with only shoes and stockings and a gingham mother Hubbard dress extending fro mthe waist down and with a light piece of cheese cloth covering the shoulders and breast. They were brought in one by one, weighed, and their height was taken on tested scales. This was done by the nurse, after which they came to me, and in every case I proceeded as follows:

1. Scalp examination for scars, eruptions, and the presence of lice.

2. Eyes examined for evidence of jaundice, equality of the pupils, reaction of the pupils, scars showing previous disease, and signs of Grave's disease.

3. Ears for discharge, or evidence of deafness.

4. Mouth: Evidence of anemia was looked for, and the condition of the teeth was carefully noted. Condition of tonsils was also looked into.

5. The neck was examined for evidenec of glandular, especially thyroid enlargement.

6. Upper extremities were examined for nervous tremor, and the pulse rate was also noted. In all save fifteen (sixty-one), the blood pressure, both systolic and diastolic, was recorded.

7. The chest, absolutely bare, was examined carefully for evidence of heart or lung disease, both front and back.

8. The abdomen was was examined for enlargement of the liver and spleen, and areas of tenderness or muscle spasm. The lower extremities were examined for signs of edema, tibial thickening, and the patella reflexes were noted.

9. Glands: The neck, the arm above the elbow, axillae, and groins were palpated for evidence of glandular enlargement.

10. Skin: Careful observation was made for the evidence of eruptions, scars, or pigmented areas showing previous eruptions.

In all cases where I found rapid pulse, distinct undernourishment, history of cough or blood spitting, or abnormal signs in the heart or lnungs, I reexamined at a later time. There were twenty-five such reexaminations. In each case after I had completed the foregoing, the girl was sent to Doctor Hodge, who made the following examination:

1. Breasts were examined for lumps, hypertrophy, or signs of pregnancy.

2. External genitalia were examined for scars, condylomata, the condition of the vaginal and urethral orifices, Bartholin's glands, and the presence of discharge. Any hypertrophy of the clitoris or internal labia was also noted.

3. Inguinal lymph nodes were examined, as an index to previous or active imflammatory processes in the lower genitourinasy tract.

The urethra was then stripped with the rubber covered finger, a sterile swab was inserted into the urethra, and smears were made from the secretions on clean glass slides. These smears were made in duplicate and were later stained and examined microscopically for the presence of gonococcus.

After the examination above described, five c. c. of blood was drawn from the vein at the elbow, for a Wassermann test. Each specimen was put at once into a sterile tube marked with the girl's name. Later a slip accompanying each tube was written out, embodying any data obtained from the girl's history or examination pointing to presence of venereal disease and any treatment the girl might have received for such a condition. The tubes and corresponding slips were then promptly sent out to the City Hospital at Bay View, where a Wassermann test for syphilis was done on each specimen, under the supervision of the resident physician, Dr. Thomas P. Sprunt.

I maght say that a very careful history was taken by Doctor Hodge of the menstrual and urinary functions and that she also inquired of every girl as to a history of pregnancy in the past, primary sores, skin eruptions, or leucorrheal discharge, while I obtained simply a history of illness occurring within five years and did not attempt to obtain facts relative to diseases within the family. I might say that the superintendent of the home had told us that all the girls were well, save three who were supposed to have minor ailments.

On acount of the fact that many of the girls had been committed by the courts to this school on account of immorality, we expected to find quite a large percentage suffering from the venereal diseases, and for that reason and as a matter of clinical interest, the girls were divided into two groups, the first group containing fifty-two girls showing no evidence on physical examination of a past syphilitic infection, and the second group containing twenty-four having one or more signs suggestive of syphilis, such as pigmented scars (fifteen girls), dental changes (nine), high blood pressure (three), and general glandular enlargement (eleven). The report on the Wassermann tests of these seventy-six girls was positive in the case of six, showed very slight fixation in seven, and no fixation in sixty-three. The six positive Wassermanns all occurred in the group of girls who showed absolutely no physical signs of a past syphilitic infection, and in the seven negative cases with slight fixation, only two had been in any way suspected of having had syphilis, one (aged twelve years) on account of her having serrated edges to her lower incisors, and the other one (seventeen years old) was suspcted on account of corrugations over front or lower incisors.

The nourishment of the girls was apparently normal, and is borne out by the fact that the total weight of those fifteen years of age and over (sixty-four) was 7,057 pounds, while the normal weight for a similar group of white women according to the medicoacturial investigation would be 7,051 pounds, a difference of only six pounds in 7,000, providing that an allowance of three pounds is made for the difference in the weight of clothing, as in the case of these girls they did not have on underclothes, corsets, or underskirts, and the dresses were below the average weight. In the medicoactuarial investigation the weights were of women applying for insurance, normally dressed, who applied during the years 1885 to 1900 inclusive, at which time the clothing of women weighed considerably more than it does now. Unfortunately, there is no table of weights that I know of for the colored race, and this may beb of importance, as it is known that Austrians and Germans of the same age and height are ten per cent. heavier than Americans, and the Japanese are ten per cent. lighter (Medico-Actuarial Investigation, 1).

The average age of those examined was sixteen and one-half years, the youngest being eleven years old, the oldest twenty-one years old lacking a few days. The average length of stay in the school was two years and two months, the shortest being a few days, while the longest stay was eight years.

The most important thing found in our investigation was the extremely bad condition of the back teeth, not noticeable on ordinary casual inspection, but readily seen when the mouth was open and the teeth were examined. Thirty-one out of the seventy-six girls had one or more teeth which were simply shells or roots. It is noteworthy that only three showed bad front teeth, which might account for the popular impression that the negro race has better teeth than the white.

In only three of the girls were the tonsils in need of treatment.

Only one girls wore glasses; none had any evidence of eye disease, and only one showed evidence of previous eye disease (corneal scar).

None of the girls had discharging ears or noteworthy deafness.

Five girls showed moderate but definite enlargement of the thyroid gland. In one it was accompanied by tremor of the fingers and rapid pulse, and in one by rapid pulse but no other sign of Grave's disease.

Two of the girls had definite compensated valvular disease of the heart, and one had a slightly enlarged heart with functional type of murmur. One of these girls had had definite inflammatory rheumatism in the past, and the other one gave a history of repeated attacks of sore throat.

The blood pressure was found to be high in one nineteen year old girl (systolic 165 mm., diastolic 110 mm.), two others having moderately high blood pressure (scystolic 144 mm. and 142 mm.).

Tuberculosis of the lungs was found in six girls, one of them having an extensive infiltration of the lung complicated by a tuberculosis peritonitis, and possibly by Pott's disease. The other five had the disease in its incipient stage with slight or no symptoms, and only very moderate physical signs. In addition, there were three girls who should be sent to the Municipal Tuberculosis Hospital (together with the other six) for observation on account of their having either suspicious symptoms or indefinite signs.

Abdominal examination showed that one girl had a small umbilical hernia, which however, did not require treatment. There were six girls who had more or less tenderness in one or both ilias fossae, unaccompanied by muscle spasm, a condition not infrequent in young girls.

There was a posisbility of one of the recently admitted girls being pregnant on account of the history she gave and the delayed menstrual period.

Three of the girls showed scars in the neck typical of old broken down tuberculosis glands of the neck, and three of the girls were found to have tuberculosis glands of the neck, which, however, did not require surgical treatment.

The results of the examinations by Dr. Mary Hodge may be briefly summarized in two groups.

a. Based on statements: 1. Urinary. Pain and burning in three cases, all giving negative urethral smears. In one of these cases a urethral caruncle was suspected.

2. Menstrual. Menstruation had not yet begun in fourteen girls, of whom one was sixteen years old, four were fifteen years old, and the rest younger. Pain was present at the periods in thirty-two cases, in only five to a considerable degree. In three cases periods had been missed for several months, ranging from four months to one year.

3. A child had been borne in one case. This girl had gonorrheal organisms in the urethral smears.

4. A miscarriage was noted in one case. In this case both the Wassermann reaction and smears were negative.

5. Pregnancy (early) was suspected from the history in one case. The girl had a purulent leucorrheal discharge, with gonorrheal organisms.

6. Venereal history suggestive of syphilis was given in four cases, of which three had negative Wassermann reactions. Two of these negative tests were explained by previous treatment with salvarsan.

b. Based on physical examination and laboratory finding: 1. Breasts were markedly hypertrophied in four cases.

2. Labia or clitoris hypertrophied in twelve cases.

3. Outlet or vagina marital in all cases.

4. Condylomata or other signs of secondary syphilis were found in no case.

5. Scars were not found on the external genitalia.

6. Discharge from the vagina was noted in twenty cases, in most of them slight; purulent and profuse in only one case.

7. Enlarged inguinal glands were found in seventeen cases. In seven of these syphilis was proved by the Wassemann test: (One of these cases showed a negative Wassermann at this date, but had had a positive test a year ago and had been treated at the City Hospital with salvarsan.) In four cases gonorrhea was proved by the finding of organisms in the smears.

8. Urethral smears were found positive, i. e., showed gonorrheal organisms, in eight cases. In all these cases the infection was in the chronic stage.

9. Wassermann tests were positive, i. e., showed active syphilis in the body, in six cases. In two more there was a definite history of syphilis, but the Wassermanns were negative after active treatment with salvarsan.

CONCLUSIONS.

We have as a basis for this study the primary fact that these girls were all considered to be healthy, and were resident of a semiprivate institution under city supervision having an attending physician, but no medical history or physical examination had been made of any of these girls, either immediately prior to their admission or during their stay in the institution. This examination has disclosed the following cases of disease.

1. Syphilis. In this group of supposedly healthy girls there are six girls who are suffering from latent syphilis, without physical signs or symptoms, and two other girls have had syhpilis in the past and have been treated with salvarsan.

2. Gonorrhea. Eight girls were shown to be suffering from chronic gonorrhea, as proved by the finding of the organism in smears. Twelve others had a leucorrheal discharge, which on one examination only failed to show gonococci. No doubt some of these girls could be shown to have gonorrhea if many smears were made.

3. Valvular heart disease. Two girls had valvular heart disease, while a third had a slightly dilated heart.

4. Tuberculosis. Six girls were found to have tuberculosis of the lungs, five in the incipient stage, and one in the advanced stage. In addition, three were suspected of having incipient tuberculosis on account of symptoms or physical signs, or both. Three girls were found to have tuberculous glands of the neck, and three girls also had scars in the neck indicative of previous tuberculous adenitis.

5. Hyperthroidism. One girl had a definite mild case of this disease, and one other was suspected of having it on account of thyroid enlargement accompanied by tachycardia.

6. Hypertension. There was one girl with this condition, and two others whose blood pressure readings were relatively high.

7. Dental caries. Thirty-one girls had teeth badly in need of attention, but in none had there been symptoms or at least complaint, and in only three was the condition suspected of casual inspection.

8. Wassermann test. This test showed six girls (eight per cent.) to have syphilis, and yet none of these girls showed any of the physical signs usually considered to be associated with syphilis (save enlarged inguinal glands), and from none of these six was there obtained a history suggestive of syphilis. This seems to confirm the growing belief among physicians that syphilis may be present and yet give rise to no physical signs which would cause one even to suspect it.

4 East Preston Street.

### SANITARY CONDITION OF BOTTLED WATERS.

The Bureau of Chemistry for several years has been investigating the sanitary conditions in the production and distribution of bottled mineral and table waters, which are offered for sale in interstate commerce and therefore subject to the Food and Drugs Act. It is recognized that the sale of bottled waters is dependent upon the belief by the public in the purity of the product. The Bureau has recently conferred with a large number of sanitary experts and bacteriologists regarding a desirable standard for judging the sanitary character of bottled waters. As a result of the investigational work and the above mentioned conferences the Bureau believes that the tolerances established by the Public Health Service of the Treasury Department for waters served on interstate carriers is none too rigid for application to bottled waters sold in interstate commerce or imported from foreign countries. The Treasury Department standards are as follows:

1. The total number of bacteria developing on standard agar plates, incubated 24 hours at 37 degrees C., shall not exceed 100 per cubic centimeter; provided, that the estimate shall be made from not less than two plates, showing such numbers and distribution of colonies as to indicate that the estimate is reliable and accurate.

2. Not more than one out of five 10 cc. portions of any sample examined shall show (by the method of the Public Health Service) the presence of organisms of the bacillus coli group.

---

### PELLAGRA PREVENTION.

### SPRING DIET DETERMINES SUMMER SYMPTOMS.

A faulty or restricted diet at this season of the year is the chief factor in the production of pellagra. Measures to prevent the development of the disease should be instituted during the early spring months, according to a circular of information issued today by the United States Public Health Service. While the manifestations of pellagra are in most cases not in evidence until June or July, the condition invariably dates from a faulty diet of earlier months. Therefore, if due precautions are exercised by individuals at the present time the havoc wrought by this scourge may be greatly lessened, if not entirely eliminated.

DANGER SIGNALS.

The report further calls attention to certain danger signals which should be recognized by those who reside in pellagrous districts or those who have had previous attacks of the disease. Among such warning symptoms are extreme nervousness or change in the mental characteristics of the individual. Weakness or debility, a disinclination to undertake the ordinary daily tasks, and unexplained digestive symptoms may all be premonitory signs. These symptoms do not, of course, necessarily mean the development of pellagra, but taken in connection with the history of a one-sided, monotonous diet, they serve as a definite warning of the possibilities of its onset.

SPRING DIET.

The diet recommended by the health service for the prevention of pellagra will not produce results if followed for a week or ten days, but if continuously and consistently used, under circumstances similar to its administration in the various institutions where the experimental tests have been performed, it will protect the individual against the development of the disease. Necessarily, a rigid unvaried diet is wholly undesirable and the menu recommended is only to indicate in a general way the character of the food to be prescribed. Frequently the element of poverty, inaccessibility to market supplies, or even personal idiosyncrasy, may require some modification of the diet table, so that strict adherence to its components may not in all respects be practicable. The object of the diet as submitted is to minimize the consumption of the carbo-hydrate (starchy and sweet) foods and to increase the amount of fresh animal protein and of fresh legumes (peas and beans).

The breakfast, for example, should consist of oatmeal and cream, without sugar, with either ham or breakfast bacon and two eggs. Not more than two thin slices of whole wheat bread should be taken, preferably untoasted. Hot bread or biscuits are inadvisable. A glass of fresh milk is to accompany the breakfast and either oranges or grape fruit may be the initial course. The dinner should consist of either pea or bean soup, prepared from dried peas or beans, with a meat stock. The meat may be beef, pork, ham, chicken, veal or mutton, prepared in whatever manner is the most appetizing, preference being given to roasting or broiling rather than frying. Hamburger steak, meat hash, or fish may be substituted to afford variety. Care should be exercised that the meats are not overdone. Of vegetables, Irish potatoes, boiled in the jacket or baked, cabbage, turnip or mustard greens, collards and lettuce, are to be recommended. For dessert, stewed, fresh or dried fruit will prove sufficient. The dinner should be accompanied by not more than two thin slices of whole wheat bread and a glass of buttermilk. The supper should consist of pork and beans, or baked beans properly seasoned, the usual amount of bread and a glass of buttermilk. If preferred, eggs, scrambled or otherwise prepared, may be substituted for the more substantial ingredient of the meal.

### DIET CHEAP AND AMPLE

A diet such as the above is not prohibitive as to cost, at least to but few of the residents of the country, affords a sufficient number of heat units, if taken in reasonable quantity, and will effectually prevent the development of a disease which alone caused 8,000 deaths in the United States during the past year.

---

### TO ORGANIZATIONS INTERESTED IN OUR FOOD SUPPLY.

Press reports and government inquiries indicate that creameries and dairies are in a frightfully unsanitary condition; that transportation and other conditions cause dairy products to be sold which are almost beyond belief· Twenty per cent of our cows are tuberculous (and the germs are creating human tuberculosis and making tubercular people). The highest U. S. Government authority says 15,000 humans die every year from this contamination. This does not take into account many other diseases which are spread from the same source.

We are not awake to this terrible danger. I do not know its full portent. So I have asked congress to investigate the matter and to report the facts. Will you help me in getting a full investigation of conditions surrounding the marketing of our dairy products, by passing a resolution asking your congressman and senators to take an active interest in determining the true state of affairs? Send them your resolution at once and I would appreciate it if you would send me a copy.

Yours sincerely,
J. CHAS. LINTHICUM,
Member of Congress.

